# Association between periodontitis and cardiovascular mortality, all-cause mortality in patients with congestive heart failure: NHANES 2009–2014

**DOI:** 10.1097/MD.0000000000046054

**Published:** 2025-12-26

**Authors:** Fenglei Guan, Tianjie Lai, Guangan Luo, Yunxian Chen, Yuanlin Zhao, Konghe Hu, Liangqiu Tang

**Affiliations:** a Department of Cardiology, The Affiliated Yuebei People’s Hospital of Shantou University Medical College, Shaoguan, P.R. China; b Department of Spine Surgery, The Affiliated Yuebei People’s Hospital of Shantou University Medical College, Shaoguan, P.R. China.

**Keywords:** all-cause mortality, cardiovascular-mortality, CHF, congestive heart failure, periodontitis

## Abstract

Periodontitis is highly prevalent worldwide, but its association with cardiovascular mortality and all-cause mortality in patients with congestive heart failure (CHF) has not yet received sufficient attention. In this study, weighted logistic regression was employed to examine the relationship between periodontitis and CHF, and weighted Cox regression was used to analyze the relationship between periodontitis and all-cause mortality and cardiovascular mortality. Subgroup analyses were conducted to investigate the impact of potential factors on the associations between periodontitis and CHF, all-cause mortality, and cardiovascular mortality. Sensitivity analyses were performed to enhance the robustness of the study results. In unadjusted and adjusted models for various covariates, significant positive associations were found between periodontitis and CHF, all-cause mortality, and cardiovascular mortality, with statistical significance. In the fully adjusted Model 4, the association between periodontitis and CHF was odds ratio: 2.763 (95% confidence interval [CI]: 1.563, 4.887), with all-cause mortality, was hazard ratio: 1.202 (95% CI: 1.047, 1.380), and with cardiovascular mortality was hazard ratio: 1.529 (95% CI: 1.181, 1.980). Periodontitis raises the risk of CHF, all-cause mortality, and cardiovascular mortality. The increased risk of CHF due to periodontitis may subsequently elevate the risks of all-cause and cardiovascular mortality.

## 1. Introduction

Periodontitis, a chronic multifactorial inflammatory disease triggered by an imbalance in oral microbiota, is characterized by subgingival infections causing local inflammation, leading to progressive destruction of the tooth-supporting apparatus, clinical attachment loss, alveolar bone loss, periodontal pocket formation, and gingival bleeding.^[1]^ As the sixth most common human disease, the global prevalence of periodontitis is as high as 45% to 50%, with severe cases affecting 11.2% of the global population.^[2]^ Extensive research has demonstrated that periodontitis is not merely a local problem but can also enter the bloodstream via bacteria and inflammatory mediators, triggering systemic immune-inflammatory responses.^[3,4]^ Increasing research also reports that periodontitis is closely related to the development of chronic diseases in various systemic organs, including metabolic syndrome, cardiovascular, liver, and kidney diseases.^[5–10]^ Congestive heart failure (CHF), as a complex clinical syndrome, is triggered by various cardiovascular diseases and is influenced or exacerbated by multiple systemic non-cardiovascular conditions.^[11,12]^ Currently, the incidence and mortality rates of HF remain high. With the aging population and the increase in chronic diseases such as coronary artery disease and hypertension, the incidence and treatment costs of HF are expected to continue rising.^[13,14]^ Since Mattila et al^[15]^ reported the first study on the relationship between dental health and myocardial infarction in 1989, more and more research has recognized the importance of co-morbid management between periodontitis and CHF. For example, Wagner^[16]^ and Sebring^[17]^ reported that improving oral health can reduce the incidence of adverse cardiovascular events.

Finally, given the high prevalence and potential inflammatory mechanisms of periodontitis, it has emerged as a significant reversible risk factor for CHF mortality. Nevertheless, the role of periodontitis in the risk of cardiovascular and all-cause mortality in HF remains underexplored. This study aims to investigate the association between periodontitis and HF by examining the period health of HF patients, assessing the independent role of periodontitis as a potential risk factor for HF, and offering new perspectives and strategies for the comprehensive management of these two conditions.

## 2. Materials and methods

### 2.1. Data source and study population

This study is based on data from the National Health and Nutrition Examination Survey (NHANES), conducted by the Centers for Disease Control and Prevention (CDC) and the National Center for Health Statistics (NCHS), which aims to comprehensively assess the health and nutritional status of the US population. This study strictly followed the Strengthening the Reporting of Observational Studies in Epidemiology Guidelines and was approved by the NCHS Research Ethics Review Board, with all participants providing written informed consent. This study analyzed NHANES datasets from 2009 to 2014.

### 2.2. Definitions of periodontitis

In defining periodontitis, according to the NHANES 2009 to 2014 standards, the diagnosis of periodontitis is based on the measurement of clinical attachment loss (CAL) and probing depth (PD). Measurements were taken at 6 sites per tooth (mesiobuccal, midbuccal, distobuccal, mesiolingual, midlingual, and distolingual) using a Hu-Friedy PCP-2 periodontal probe, recording gingival recession and PD, and calculating CAL. According to the classification standards of the CDC and the American Academy of Periodontology, mild periodontitis is defined as having two or more interproximal sites with attachment loss (AL) ≥ 3 mm and two or more interproximal sites with PD ≥ 4 mm (not on the same tooth), or one interproximal site with PD ≥ 5 mm. Moderate periodontitis involves two or more interproximal sites with AL ≥ 4 mm, or two or more interproximal sites with PD ≥ 5 mm (not on the same tooth). Severe periodontitis is defined as having two or more interproximal sites with AL ≥ 6 mm (not on the same tooth) and at least one interproximal site with PD ≥ 5 mm. Thus, the patient is diagnosed as “non-periodontitis” when neither CAL nor PD reaches the thresholds defined by the CDC/American Academy of Periodontology case definition.

### 2.3. Definition of CHF

The definition of CHF primarily relies on participants’ self-reports and clinical assessments. Specifically, in the NHANES screening, CHF is defined as a “yes” response to the question, “Has a doctor or health professional ever told you that you have CHF?” Participants must be 20 years or older and have complete relevant data available for analysis. To further enhance the accuracy of CHF diagnosis, NHANES combines self-reported data, clinical assessments, and review of medical records.

Exclusion criteria include: age under 20 years; incomplete self-reported and clinical assessment data; no available medical records or SUA data; and incomplete mortality data.

### 2.4. Covariates considered

Several key covariates were included to account for potential confounders adequately. These covariates encompass sociodemographic characteristics, lifestyle habits, physical measurements, and specific health conditions. Specifically, this includes age, sex, race (non-Hispanic Black, non-Hispanic White, other/mixed race, Mexican American, and other Hispanic), body mass index (BMI, in kg/m^2^), level of physical activity (calculated by weekly time and intensity), smoking status (never smoked, former smoker, current smoker), alcohol consumption (categorized as never drank, former drinker, light drinker, moderate drinker, and heavy drinker), income level (dichotomized as poor and non-poor), and education level (ranging from less than 9th grade to college and beyond). Additionally, we included triglycerides (measured in mg/dL) and cholesterol (measured in mg/dL) as important physiological indicators. The presence of 2 common diseases, cancer and diabetes, was also included and expressed as prevalence percentages.

### 2.5. Statistical analysis

In this study, we took into account the complex multistage, four-stage sampling design used by NHANES. According to official recommendations, we included sample weights, stratification, and clustering in all statistical analyses, strictly following NHANES analysis and reporting guidelines. Furthermore, we classified the baseline characteristics of participants in detail according to whether they had periodontitis. We reported means and 95% confidence intervals (CIs) for continuous variables, while we used percentages with 95% CIs for categorical variables. To compare differences between groups, we used the Wilcoxon rank-sum test for continuous variables, and for categorical variables, we used the chi-square test with Rao & Scott second-order correction to more accurately address the complexity introduced by the sample design.

We utilized weighted logistic regression to assess the linear association between periodontitis and CHF, presenting the strength of association as odds ratio and its 95% CIs. We employed weighted Cox proportional hazards regression to examine the association between periodontitis and all-cause and cardiovascular mortality. We developed 4 models, employing the following strategy for covariate selection in Models 2 and 3: Introduce covariates into the basic model or exclude them from the full model if they change the explanatory variable’s regression coefficient by more than 10%. Conduct univariate analysis where the *P* value of the covariate’s regression coefficient for the outcome variable is less than .1. Any covariate meeting either criterion was defined as a key covariate and included in the model. The specific adjustments for the models were as follows: Model 1 did not adjust for covariates; Model 2 adjusted for age, sex, smoking status, hypertension, and educational attainment, which are key covariates in the association between periodontitis and all-cause and cardiovascular mortality. Model 3 adjusted for age, income level, educational attainment, alcohol use, smoking status, BMI, cancer, diabetes, triglycerides, cholesterol, and hypertension. These factors are key covariates in the association between periodontitis and CHF. Model 4 adjusted for all considered covariates: age, sex, race, income level, educational attainment, alcohol use, smoking status, BMI, activity level, cancer, diabetes, triglycerides, cholesterol, and hypertension.

In addition to adjusting for covariates such as age in all multivariable models, we also conducted stratified analyses, including subgroup analyses by age (<60 years and ≥60 years), to explore the potential impact on the associations between periodontitis and CHF, as well as all-cause and cardiovascular mortality. To strengthen the robustness of our conclusions, we also performed sensitivity analyses, merging no and mild periodontitis into one group and moderate and severe periodontitis into another group for statistical analysis. A two-tailed *P* < .05 was considered statistically significant in all analyses, and all statistical analyses were conducted using R software version 4.3.1.

### 2.6. Ethics approval and consent to participate

This study adhered to the ethical standards of the Helsinki Declaration and its amendments or equivalent standards. The data used were sourced from the NHANES survey approved by the NCHS Ethics Review Board. As this research involved secondary analysis of the NHANES database and complied with Strengthening the Reporting of Observational Studies in Epidemiology guidelines, no additional ethical approval was required. For detailed NHANES methodology and ethical standards, visit the CDC and NCHS websites: https://www.cdc.gov/nchs/nhanes/irba98.htm.

## 3. Results

### 3.1. General characteristics of participants (Table 1)

This study included 12,236 participants. According to Table 1, the average age of participants with periodontitis was 53 years, compared to 43 years for those without periodontitis. Given this clear age difference, we adjusted for age in all regression models and also performed age-stratified subgroup analyses (Tables 3 and 4), which confirmed the robustness of the associations. Participants with periodontitis were generally male, with higher BMI, higher physical activity levels, and elevated triglycerides and cholesterol levels. Furthermore, these individuals were more likely to consume alcohol and smoke, had lower income and education levels, and exhibited higher rates of cancer and diabetes.

**Table 1:** General characteristics of participants.

Characteristic	Periodontitis (N = 12,236)		*P* value†
	No, N = 8278*	Yes, N = 3958*	
Age, yr	43 [42, 43]	53 [52, 54]	<.001
BMI, kg/m^2^	27.9 [28, 28]	28.8 [29, 29]	<.001
Physical activity (activity level × min/wk)	1723 [1601, 1845]	3737 [3495, 3978]	<.001
Triglycerides, mg/dL	138 [135, 142]	166 [157, 175]	<.001
Cholesterol, mg/dL	198 [197, 199]	200 [198, 202]	.026
Sex, %			<.001
Male	51 [49, 52]	63 [62, 65]	
Female	49 [48, 51]	37 [35, 38]	
Race, %			<.001
Non-hispanic black	7.3 [6.3, 8.4]	14 [12, 16]	
Non-hispanic white	78 [76, 80]	65 [61, 69]	
Other/multiracial	4.1 [3.5, 4.8]	6.5 [5.3, 8.0]	
Mexican American	5.5 [4.6, 6.7]	9.3 [7.3, 12]	
Other hispanic	5.0 [3.6, 6.9]	5.8 [4.6, 7.4]	
Alcohol use, %			<.001
Never	10.0 [7.6, 13]	9.3 [8.1, 10]	
Former	12 [11, 13]	18 [16, 19]	
Mild	39 [36, 41]	35 [33, 38]	
Moderate	19 [18, 20]	15 [13, 17]	
Heavy	21 [19, 22]	23 [21, 25]	
Income level, %			<.001
Poor	8.8 [7.7, 9.9]	15 [13, 17]	
Not poor	91 [90, 92]	85 [83, 87]	
Smoke status, %			<.001
Never	58 [55, 60]	41 [39, 43]	
Former	23 [22, 25]	30 [28, 32]	
Now	19 [17, 21]	29 [27, 32]	
Education attainment, %			<.001
Less than 9th grade	2.5 [2.1, 3.0]	6.7 [5.7, 7.9]	
9–11th grade	7.4 [6.6, 8.3]	15 [13, 16]	
High school grade/GED	22 [20, 24]	27 [25, 29]	
Some college or AA degree	31 [30, 33]	30 [28, 32]	
College graduate or above	36 [34, 39]	22 [20, 24]	
Cancer, %	6.7 [6.0, 7.4]	9.6 [8.3, 11]	<.001
Diabetes, %	4.9 [4.4, 5.5]	11 [10, 13]	<.001

BMI = body mass index, CI = confidence interval, GED = general educational development.

* Mean; %.

† Wilcoxon rank-sum test for complex survey samples; chi-squared test with Rao & Scott’s second-order correction.

**Table 2:** Association between different CHF and all-cause mortality, cardiovascular mortality.

Model	Characteristic	CHF			All-cause mortality			Cardiovascular mortality		
		OR	95% CI	*P* value	HR	95% CI	*P* value	HR	95% CI	*P* value
Model 1	Periodontitis									
	No	Ref	Ref		Ref	Ref		Ref	Ref	
	Yes	6.036	3.813, 9.555	<.001	3.192	2.747, 3.710	<.001	4.214	3.175, 5.594	<.001
Model 2	Periodontitis									
	No	Ref	Ref		Ref	Ref		Ref	Ref	
	Yes	2.740	1.665, 4.510	<.001	1.219	1.060, 1.401	.005	1.515	1.186, 1.934	<.001
Model 3	Periodontitis									
	No	Ref	Ref		Ref	Ref		Ref	Ref	
	Yes	2.442	1.466, 4.069	<.001	1.229	1.077, 1.404	.002	1.596	1.232, 2.067	<.001
Model 4	Periodontitis									
	No	Ref	Ref		Ref	Ref		Ref	Ref	
	Yes	2.763	1.563, 4.887	<.001	1.202	1.047, 1.380	.009	1.529	1.181, 1.980	.001

Model 1: Not adjusted.

Model 2: Adjusted age, sex, smoke status, hypertension, education attainment.

Model 3: Adjusted age, income level, education attainment, alcohol use, smoke status, BMI, cancer, diabetes, triglycerides, cholesterol, hypertension.

Model 4: Adjusted age, sex, race, income level, education attainment, alcohol use, smoke status, BMI, activity level, cancer, diabetes, triglycerides, cholesterol, hypertension.

BMI = body mass index, CHF = congestive heart failure, CI = confidence interval, HR = hazard ratio, OR = odds ratio.

**Table 3:** Subgroup analysis of the association between periodontitis and CHF.

Character	No periodontitis	Periodontitis‡	*P* value§	*P* for interaction
Age strata				.485
Young to middle-aged*	ref	4.094 (1.710, 9.804)	.002	
Older†	ref	2.589 (1.301, 5.152)	.007	
Sex				.535
Male	ref	3.236 (1.469, 7.126)	.004	
Female	ref	2.256 (0.975, 5.217)	.057	
Hypertension				.418
No	ref	4.518 (1.435, 14.223)	.011	
Yes	ref	2.390 (1.420, 4.023)	.001	

CHF = congestive heart failure, CI = confidence interval, OR = odds ratio.

* Age < 60.

† Age ≥ 60.

‡ OR (95% CI) for CHF.

§ Adjusted for all covariates.

**Table 4:** Subgroup analysis of the association between periodontitis and cardiovascular mortality and all-cause mortality.

Outcome	Character	No periodontitis	Periodontitis‡	*P* value§	*P* for interaction
Cardiovascular mortality	Age strata				<.0001
	Young to middle-aged	ref	1.947 (1.556, 2.436)	<.0001	
	Older	ref	1.231 (1.019, 1.486)	.031	
	CHF				.854
	No	ref	1.184 (1.022, 1.372)	.025	
	Yes	ref	1.004 (0.528, 1.910)	.990	
All-cause mortality	Age strata				<.001
	Young to middle-aged*	ref	2.990 (1.769, 5.054)	<.0001	
	Older†	ref	1.534 (1.166, 2.018)	.002	
	CHF				.83
	No	ref	1.493 (1.132, 1.968)	.004	
	Yes	ref	1.586 (0.318, 7.914)	.574	

CHF = congestive heart failure, CI = confidence interval, OR = odds ratio.

* Age < 60.

† Age ≥ 60.

‡ OR (95% CI) for cardiovascular mortality and all-cause mortality.

§ Adjusted for all covariates.

### 3.2. Association between different CHF and all-cause mortality, cardiovascular mortality (Table 2)

In this study, we employed weighted multivariate logistic regression to assess the association between periodontitis and CHF. We weighted Cox regression to examine the relationship between periodontitis and all-cause as well as cardiovascular mortality. These findings are shown in Table 2. The analysis indicated that periodontitis was significantly positively associated with CHF, all-cause mortality, and cardiovascular mortality in both unadjusted and adjusted models for various covariates, with statistical significance. In Model 4, which adjusted for all covariates, the association between periodontitis and CHF was odds ratio: 2.763, 95% CI: (1.563, 4.887), the association with all-cause mortality was hazard ratio: 1.202, 95% CI: (1.047, 1.380); and the association with cardiovascular mortality was hazard ratio: 1.529, 95% CI: (1.181, 1.980). This indicates that, compared to participants without periodontitis, those with periodontitis had a 176.3% increased risk of developing CHF, a 20.2% increased risk of all-cause mortality, and a 52.9% increased risk of cardiovascular mortality.

### 3.3. Subgroup analysis of the association between periodontitis and CHF (Table 3)/subgroup analysis of the association between periodontitis and cardiovascular mortality and all-cause mortality (Table 4)

We performed a stratified subgroup analysis (Table 3) to investigate the potential factors affecting the association between periodontitis and CHF and to examine the effect of CHF on the relationship between periodontitis and all-cause mortality and cardiovascular mortality (Table 4). The results in Table 3 indicate that the association between periodontitis and CHF is robust (*P* for interaction < .05), with statistically significant differences observed across different age groups, genders, and hypertension status (*P* < .05). The results in Table 4 show that the association between periodontitis and the risks of all-cause mortality and cardiovascular mortality is influenced by age (*P* for interaction < .05), with this positive association being more pronounced in young to middle-aged populations. Additionally, the association between periodontitis and all-cause and cardiovascular mortality is statistically significant in individuals without CHF, suggesting that periodontitis may increase the risk of CHF, thereby raising the risk of all-cause and cardiovascular mortality.

### 3.4. Sensitivity analysis of the association between periodontitis and cardiovascular mortality and all-cause mortality

We conducted sensitivity analyses to ensure the reliability of the results, merging patients with no and mild periodontitis into one group and those with moderate and severe periodontitis into another group. The statistical analysis showed that the relationship between periodontitis and CHF, all-cause mortality, and cardiovascular mortality was consistent with the previous analysis, reinforcing the robustness of the study results. Detailed results can be found in Table S1, Supplemental Digital Content, https://links.lww.com/MD/Q790.

## 4. Discussion

Our study clarified the significant positive associations between periodontitis and CHF, all-cause mortality, and cardiovascular mortality. After adjusting for multiple covariates, the results showed that periodontitis patients had significantly increased risks of CHF, all-cause mortality, and cardiovascular mortality.

Whether the increased mortality risk from HF associated with periodontitis is due to a biological link or shared risk factors remains to be explored. Previous epidemiological studies have clearly indicated a strong association between periodontitis and HF.^[18,19]^ Several studies have noted that patients with periodontitis have significantly higher levels of endothelial dysfunction, increased arterial stiffness, and elevated arterial calcification scores compared to those without periodontitis.^[20]^ Similarly, data from a health study in Hamburg City involving 6209 participants, including comprehensive echocardiography and periodontal screening, revealed a significant association between severe periodontitis and reduced and mildly reduced ejection fraction HF.^[21]^ Further research has also found that tooth loss, as an indicator of oral health, can predict adverse cardiovascular events such as HF, myocardial infarction, and stroke. Each missing tooth increases the risk of myocardial infarction, HF, and stroke.^[17,22,23]^ Interestingly, a study examining the medication-purchasing behavior of periodontitis patients found that they are more likely to require medications for cardiovascular diseases, diabetes, and other systemic conditions.^[24]^ This phenomenon seems to support further the idea that periodontitis may indirectly affect cardiovascular health by increasing the risk of systemic diseases.

Regarding microbiological mechanisms, studies have shown that periodontal pathogens such as Enterococcus faecalis and Porphyromonas gingivalis are prevalent in periodontitis patients. These bacteria can affect the cardiovascular system by inducing platelet activation, inflammation, and fibrosis.^[25,26]^ Animal studies have found prolonged exposure to Porphyromonas gingivalis lipopolysaccharides induces myocardial cell apoptosis and fibrosis, further activating adverse cardiac signaling pathways.^[27]^ The specific mechanisms may involve the oral microbiome driving autoimmune responses and the release of inflammatory markers rather than being solely linked to already established cardiovascular risk factors.^[28,29]^ Elhaieg et al^[30]^ found that, compared to control groups, histopathological examinations of periodontitis rat models showed more significant myocardial tissue damage and more commonly exhibited reduced ejection fraction. Additionally, Reina et al^[31]^ found that anti-β1-adrenergic receptor antibodies in the serum of periodontitis patients can induce cardiac cell apoptosis, potentially leading to HF.

The causative relationship is still uncertain despite observational and epidemiological studies indicating a link between periodontitis and HF. A two-sample Mendelian randomization study based on genome-wide association studies also failed to provide direct evidence that periodontitis is a cause of cardiovascular disease.^[32]^

Finally, research by Pink et al^[33]^ identified that periodontitis, in conjunction with systemic inflammation, synergistically increases the risk of cardiovascular death and all-cause mortality. Further studies have indicated that periodontitis is significantly associated with both cardiac and non-cardiac infections, particularly in patients with severe HF, where periodontal inflammation may increase infection risk, thereby affecting overall survival.^[34]^ Our study found that even after adjusting for various confounding factors in Model 4, there remains a significant association between periodontitis and both cardiovascular and all-cause mortality in HF patients. This implies that periodontitis may be a reversible mediator of cardiovascular and all-cause mortality in HF patients.

Research utilizing data from Danish nationwide registries revealed that individuals who did not have dental visits before their first myocardial infarction experienced a 47% increased risk of major adverse cardiovascular events within the next 3 years, particularly HF and cardiovascular mortality.^[16]^ Conversely, patients who received periodontal treatment had a risk of recurrent cardiovascular events comparable to those with good periodontal health. This unexpected non-causal relationship is also thought to be determined by changes in the oral microbiome and its relationship with worsening systemic inflammatory responses.^[35]^ Furthermore, periodontal therapy significantly lowers NT-proBNP and TNF-α levels in the blood of HF patients and improves vascular health metrics, such as reducing pulse wave velocity and augmentation index.^[36,37]^ Research has also shown that root canal treatment can decrease the systemic inflammatory burden, including high-sensitivity C-reactive protein and its monomeric form mCRP, in individuals with cardiovascular risk and apical periodontitis.^[38]^ Individuals with poor responses to periodontal treatment have a significantly increased incidence of cardiovascular diseases.^[39]^ Therefore, improving dental care, particularly periodontal treatment and maintenance in elderly patients, may help prevent infections in severe HF patients.^[34,40,41]^ These findings underscore the importance of periodontal health management in the secondary prevention of HF.

## 5. Strengths and limitations

There are several limitations in this study regarding the investigation of the relationship between periodontitis and HF. Firstly, while it is known that periodontitis may be related to the purchase of cardiovascular disease medications, we were unable to include the use of specific drugs like captopril due to the limited sample size. Secondly, our study subjects were predominantly from North America, so the results may not apply to populations in other regions. Thirdly, the diagnosis of HF was based on self-reported information from the participants, which may lead to the omission of some covert HF cases. Lastly, as our study is cross-sectional, the dataset does not provide information on the duration of periodontitis, making it impossible to explore the potential correlation between the duration of periodontitis and the risk of HF. These limitations suggest that we cannot draw definitive conclusions about the causality and impact between periodontitis and HF, and future research should address these issues further.

Another limitation is that we were not able to perform an age-matched comparison between participants with and without periodontitis. Given the high prevalence of periodontitis in older adults, it is difficult to identify elderly individuals who are completely free of periodontitis. Although we adjusted for age in multivariable models and performed stratified analyses, residual confounding by age cannot be entirely excluded.

## 6. Conclusions

This study used weighted regression analysis to elucidate the significant positive associations between periodontitis and CHF, all-cause mortality, and cardiovascular mortality. Adjusting for multiple covariates revealed that periodontitis patients have significantly elevated risks of CHF, all-cause mortality, and cardiovascular mortality. This indicates that periodontitis is not only an oral health issue but may also pose a severe threat to cardiovascular health. Therefore, enhancing the prevention and treatment of periodontitis is crucial for reducing the risks of CHF and cardiovascular-related mortality.

## Author contributions

**Conceptualization:** Fenglei Guan, Tianjie Lai, Konghe Hu, Liangqiu Tang.

**Data curation:** Fenglei Guan, Tianjie Lai, Guangan Luo, Yunxian Chen, Yuanlin Zhao.

**Formal analysis:** Fenglei Guan, Tianjie Lai, Yuanlin Zhao.

**Funding acquisition:** Fenglei Guan, Tianjie Lai.

**Investigation:** Fenglei Guan, Tianjie Lai, Guangan Luo, Yuanlin Zhao.

**Methodology:** Fenglei Guan, Tianjie Lai, Guangan Luo, Yunxian Chen.

**Project administration:** Fenglei Guan, Tianjie Lai, Liangqiu Tang.

**Resources:** Fenglei Guan, Tianjie Lai.

**Software:** Fenglei Guan, Tianjie Lai.

**Supervision:** Fenglei Guan, Tianjie Lai, Konghe Hu.

**Validation:** Fenglei Guan, Tianjie Lai.

**Visualization:** Fenglei Guan, Tianjie Lai.

**Writing – original draft:** Fenglei Guan, Tianjie Lai, Konghe Hu.

**Writing – review & editing:** Fenglei Guan, Tianjie Lai, Yunxian Chen, Konghe Hu, Liangqiu Tang.

## Supplementary Material


