# Supplementary material for: Association between periodontitis and cardiovascular mortality, all-cause mortality in patients with congestive heart failure: NHANES 2009–2014
Source: Medicine (Baltimore). 2025 Dec 26;104(52):e46054. doi: 10.1097/MD.0000000000046054 (PMC12746919; doi:10.1097/MD.0000000000046054)
Supplement: Supplementary file 1 [file medi-104-e46054-s001.docx]

|  | | CHF | | | All-cause mortality | | | Cardiovascular-mortality | | |
| --- | --- | --- | --- | --- | --- | --- | --- | --- | --- | --- |
| **Model**^1^ | **Characteristic** | **OR**^2^ | **95% CI**^2^ | **p-value** | **HR**^2^ | **95% CI**^2^ | **p-value** | **HR**^2^ | **95% CI**^2^ | **p-value** |
| Model 1 | Periodontitis2 |  |  |  |  |  |  |  |  |  |
|  | No/Mild | Ref | Ref |  | Ref | Ref |  | Ref | Ref |  |
|  | Moderate/Severe | 5.384 | 3.397, 8.534 | **<0.001** | 3.652 | 3.118, 4.277 | **<0.001** | 4.576 | 3.290, 6.364 | **<0.001** |
| Model 2 | Periodontitis2 |  |  |  |  |  |  |  |  |  |
|  | No/Mild | Ref | Ref |  | Ref | Ref |  | Ref | Ref |  |
|  | Moderate/Severe | 2.239 | 1.346, 3.723 | **0.002** | 1.232 | 1.071, 1.418 | **0.004** | 1.435 | 1.083, 1.900 | **0.012** |
| Model 3 | Periodontitis2 |  |  |  |  |  |  |  |  |  |
|  | No/Mild | Ref | Ref |  | Ref | Ref |  | Ref | Ref |  |
|  | Moderate/Severe | 1.973 | 1.170, 3.326 | **0.012** | 1.231 | 1.076, 1.408 | **0.003** | 1.486 | 1.104, 1.999 | **0.009** |
| Model 4 | Periodontitis2 |  |  |  |  |  |  |  |  |  |
|  | No/Mild | Ref | Ref |  | Ref | Ref |  | Ref | Ref |  |
|  | Moderate/Severe | 2.212 | 1.247, 3.925 | **0.007** | 1.209 | 1.056, 1.384 | **0.006** | 1.435 | 1.072, 1.920 | **0.015** |
| ^1^Models: Model 1 : Not adjusted Model 2 : Adjusted Age, Sex, Smoke status, Hypertension, Education attainment Model 3 : Adjusted Age, Income level, Education attainment, Alcohol use, Smoke status, BMI, Cancer, Diabetes, Triglycerides, Cholesterol, Hypertension Model 4 : Adjusted Age, Sex, Race, Income level, Education attainment, Alcohol use, Smoke status, BMI, Activity level, Cancer, Diabetes, Triglycerides, Cholesterol, Hypertension | | | | | | | | | | |
| ^2^OR = Odds Ratio, CI = Confidence Interval, HR = Hazard Ratio | | | | | | | | | | |
